# Mimicking tumor hypoxia and tumor-immune interactions employing three-dimensional in vitro models

**DOI:** 10.1186/s13046-020-01583-1

**Published:** 2020-05-01

**Authors:** Somshuvra Bhattacharya, Kristin Calar, Pilar de la Puente

**Affiliations:** 1 Cancer Biology and Immunotherapies Group, Sanford Research, 2301 E 60th Street N, Sioux Falls, SD 57104 USA; 2 Department of Surgery, University of South Dakota Sanford School of Medicine, Sioux Falls, SD USA; 3 Department of Chemistry and Biochemistry, South Dakota State University, Brookings, SD USA

**Keywords:** Tumor microenvironment, Three-dimensional, In vitro models, Hypoxia, Immune, Bioengineering, Cancer

## Abstract

The heterogeneous tumor microenvironment (TME) is highly complex and not entirely understood. These complex configurations lead to the generation of oxygen-deprived conditions within the tumor niche, which modulate several intrinsic TME elements to promote immunosuppressive outcomes. Decoding these communications is necessary for designing effective therapeutic strategies that can effectively reduce tumor-associated chemotherapy resistance by employing the inherent potential of the immune system.

While classic two-dimensional in vitro research models reveal critical hypoxia-driven biochemical cues, three-dimensional (3D) cell culture models more accurately replicate the TME-immune manifestations. In this study, we review various 3D cell culture models currently being utilized to foster an oxygen-deprived TME, those that assess the dynamics associated with TME–immune cell penetrability within the tumor-like spatial structure, and discuss state of the art 3D systems that attempt recreating hypoxia-driven TME-immune outcomes. We also highlight the importance of integrating various hallmarks, which collectively might influence the functionality of these 3D models.

This review strives to supplement perspectives to the quickly-evolving discipline that endeavors to mimic tumor hypoxia and tumor-immune interactions using 3D in vitro models.

## Background

The tumor microenvironment (TME) encompasses a complex cluster of cells that are programmed to fuel initiation, progression, and metastasis of cancer [1]. The TME is composed of tumor cells, various secreted factors and extracellular matrix (ECM), which provides structural and biochemical support to the surrounding cells [2]. The cells that constitute TME include fibroblasts, mesenchymal stem cells, macrophages, lymphocytes, endothelial cells, epithelial cells, and pericytes [3]. Collectively, these cells exert their influence on tumor progression and ECM remodeling [3]. TME secreted factors, including cytokines, integrins, proteases, and microRNAs function as signals between cells, or tools in ECM remodeling [4]. Each of these components within the tumor has unique roles and collectively hinder immune recognition and response, promote malignancy, and diminish cancer cytotoxicity within the TME [5].

**Fig. 1:**
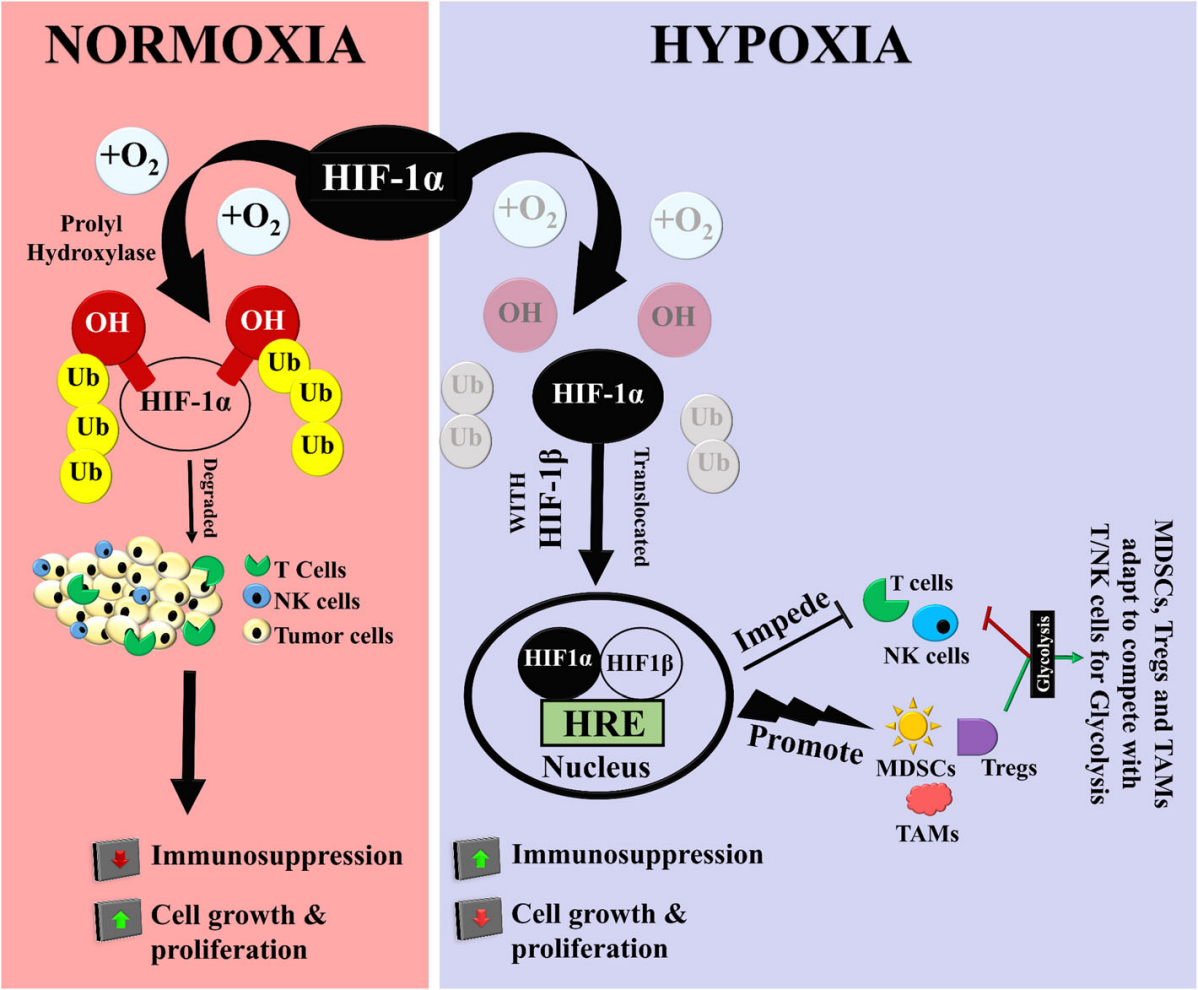
Hypoxia within TME influences tumor-immune interactions. Despite HIF-1α being generated under normal conditions, available oxygen through the effect of prolyl hydroxylase helps to degrade it ubiquitously without any detrimental downstream effects: immune participants such as NK and T cells can effectively eliminate rapidly proliferating tumor cells. When this oxygen source is deprived, as commonly seen in tumor hypoxia, degradation of HIF-1α is inhibited, leading to the translocation of HIF-1α to inside the nucleus and a cascade of events that involves the binding of HIF-1α and HIF-1β heterodimers to hypoxia-responsive elements (HREs) on DNA. The outcomes of these processes eventually impact hypoxia-responsive outcomes like incidence of pro-tumor immunosuppressant MDSCs, Tregs, and TAMs. These immunosuppressive entities adapt to the oxygen-deprived conditions and compete with T/NK cells for glycolysis and decrease functional efficacy of T cells and NK cells. The collective influence of these events results in modified cell metabolism, enhanced angiogenesis, and increased tumor proliferation and drug resistance

Hypoxia within the TME is a significant tumor feature that can influence tumor-immune interactions. Under physiological conditions where oxygen is abundantly available, the conserved proline residues of hypoxia-inducible factor 1-alpha (HIF-1α) subunits are ubiquitously degraded through hydroxylation of its alpha subunits by the oxygen-dependent enzyme HIF-prolyl-4-hydroxylases (PHDs) [6]. Following this, von Hippel–Lindau protein (pVHL), an E3 ubiquitin ligase, is bound to the hydroxylated HIF-1α hence catalyzing the proteasomal degradation of HIF-1α. During conditions of oxygen deprivation, as typically seen in hypoxia within the TME, HIF prolyl-hydroxylases are inhibited, thus perpetuating the presence of functional HIF-1α (Fig. 1) [6]. The undegraded HIF-1α is now translocated to the hypoxia-responsive elements (HREs) of its corresponding target genes, eventually upregulating their transcription [7]. As the mechanism to degrade HIF-1α is turned off, cells adapt their metabolism to function through the HIF-1α signaling biochemical cascade [8]. A direct consequence of this modulated metabolism functioning is overexpression of lactate dehydrogenases, pyruvate dehydrogenase kinases and other enzymes that regulate glycolysis. Increased expression of these enzymes leads to accelerated glucose uptake and glycolysis [9]. The effects of increased HIF-1α functioning also contribute to increased production and recruitment of pro-tumor entities such as myeloid-derived suppressor cells (MDSCs), regulatory T cells (Tregs), and tumor-associated macrophages (TAMs) which modulate the surrounding tumor niche and compete with T cells and NK cells for glycolysis [10, 11]. Studies have conclusively shown that by reversing these metabolic changes, both T-cell glycolysis and the production of IFN-γ can be significantly restored [11]. A sustained presence of undegraded HIF-1α in the hypoxic tumor core can also correlate to differentiation checkpoints for Treg or Th17 cells, and an upregulation of PD-1 ligand expression on cancer cells. Reports have shown that Th17 cells particularly favor these increased glycolytic processes associated with undegraded HIF-1α under hypoxic conditions [12]. It has also been shown that by therapeutically blocking glycolysis during the differentiation stage of Th17 cells, Treg formation can be significantly promoted [13]. The collective influence of these changes correlates to several immune evasive consequences that stimulate the TME to either resist infiltration of cytotoxic T cells and NK cells [14, 15] or to inactivate the already infiltrated immune cells in the immunosuppressive microenvironment [16]. Several studies have highlighted that these evasive immune outcomes in the TME could potentially result in increased resistance to chemo and immune therapies [17, 18]. A recently published report has shown that there is a significant correlation between weakened immune infiltration and TME hypoxic scores [14]. This study identified a link between multiple gene signatures within the TME that are specific to tumor hypoxia and lymphocyte infiltration. The results from this study indicate that reduced CD8^+^ cell cytolytic activity driven by low oxygen could be of clinical significance as a prognostic factor for various tumor types, where hypoxia-driven CD8^+^ cell cytolytic activity could be associated with worse outcomes. A significant process driving hypoxic TME to influence immunosuppression is the enhancement of anaerobic glycolysis [15]. During hypoxia-driven anaerobic glycolysis in TME, tumor-infiltrating CD8+ T cells have to compete with host tumor cells for glucose, thus ultimately inhibiting their effective response [16].

The continually evolving TME implements several biological events that influence tumor-immune interactions [19]. Physiologically, immune cells like CD8^+^ lymphocytes possess the capability to control cancer growth by exerting cytotoxic action over the cancerous tumor cells in a sustained manner [20]. However, the oxygen-deprived TME also enhances the secretion of immunosuppressive cytokines and chemokines that create a hostile environment for immune cell cytolytic activity [21, 22]. The collective influence of these components manipulates TME pathology and its role in chemoresistance [23]. Cancer cells within the tumor, along with other tumor-associated cells, control their surrounding hypoxic niche by enduring a dysfunctional metabolism leading to altered cell transdifferentiation, i.e., enhanced cancer-associated myofibroblast production [24], incorrect cytokine expressions [25], and altered ECM composition [26]. The utilization of three-dimensional (3D) in vitro models for deciphering the complex tumor immune communications yields dependable outcomes that help overcome therapeutic shortcomings [27]. In this review, we systematically review some of the current state-of-art 3D models that address the synergy between tumor hypoxia and tumor-immune interactions.

## Mimicking TME in three-dimensions

**Fig. 2:**
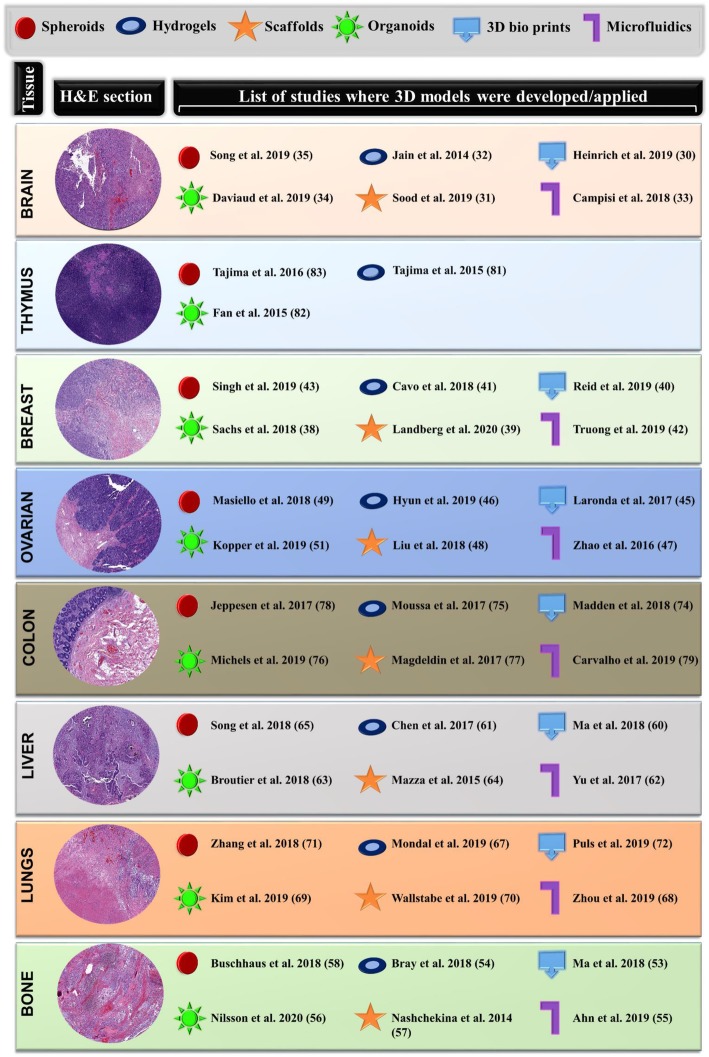
Solid tumors originating from different tissues whose micro environmental features have been replicated in 3D models. Hematoxylin and eosin (H&E) stained sections of various human solid tumors and a catalog of various types of 3D in vitro models (spheroids, organoids, hydrogels, 3D scaffolds, 3D bio printing, and microfluidics) that have been employed to regenerate the stromal tissue microenvironment in tumors of brain, breast, ovarian, bone marrow, liver, lungs, colon and thymus

The complex nature of cancer models and their ability to recreate the TME varies widely, ranging from two-dimensional (2D) models to animal-based models. Although animal models are viewed as critical platforms to test anti-tumor drugs, mouse models are costly, relatively time-consuming, and the studies performed using mice are often not repeatable in human trials [28]. Also, to decrease animal distress and mitigate ethical concerns, scientists are tasked to find alternative methods that replace, reduce, and refine the use of animals [29]. To address these issues, 3D cell culture models are a reliable alternative, providing experimentally accessible human models to study the biological processes of cancer. Several 3D culture platforms such as spheroids, organoids, hydrogels, 3D scaffolds, 3D bio-printing, and microfluidics have attempted the recreation of certain aspects of tumor microenvironments present in tissues including the brain [30–36], breast [37–43], ovarian [44–51], bone [52–58], liver [59–65], lung [66–72], colon [73–79] and thymus [80–83], as illustrated in Fig. 2. Most of these studies have employed the stromal component of the tissue’s matrix to serve as the base platform. Various approaches taken to mimic several features of the TME in 3D models include the development of tumor spheroids [84], the design of scaffold-based TME models [85], patient-derived xenograft systems where cells isolated from patients are incorporated into rodents for investigative purposes [86], organoids that can be self-organized into desirable tissue phenotypes and mimic the functionality of an organ while expressing one or more cell types [87], highly maneuverable microfluidic systems [88], and more recently 3D bio-printing platforms that can recreate the TME under highly controllable parameters yielding tailored 3D tissue architecture [89]. Although each 3D model has its pros and cons, studying disease pathology in glandular or stratified tissues has become extremely convenient using these systems [37]. For example, organoids, with their capability to spontaneously support differentiation and self-organization of their host cells [90], have efficiently yielded outstanding research breakthroughs through stunning microscopy images, revealing critical molecular and cellular mechanisms that affect disease progression [91]. Organoids have earned the title of ‘Method of the Year 2017’ [92], and since then, their applications in therapeutic testing have seen a tremendous increase. However, most of the currently available 3D models have encountered a high attrition rate in terms of clinical translatability. This is due to their inability to generate patient-specific phenotypes, thus failing to accurately predict the degree of therapy response foreseen in specific patients or generate a tangible model to clinic therapeutic efficiency amongst different patients [93]. The most significant causes of these hurdles include the inability to entirely re-engineer a heterogeneous TME [94] and the lack of an approach to mimic the native streamlined modulation caused by spatial heterogeneity [95] within 3D models. Another important reason in vitro 3D models have failed to show clinical efficacy is due to the inter-patient variability [96]. Personalized approaches that can reinstate patient-specific cues could overcome these hurdles. The complex nature of the tumor-associated stromal components, including their organization, differences in oxygen content relative to tumor volume and disease location, and the immune system composition in each patient, collectively create many challenges for scientists and clinicians.

**Fig. 3:**
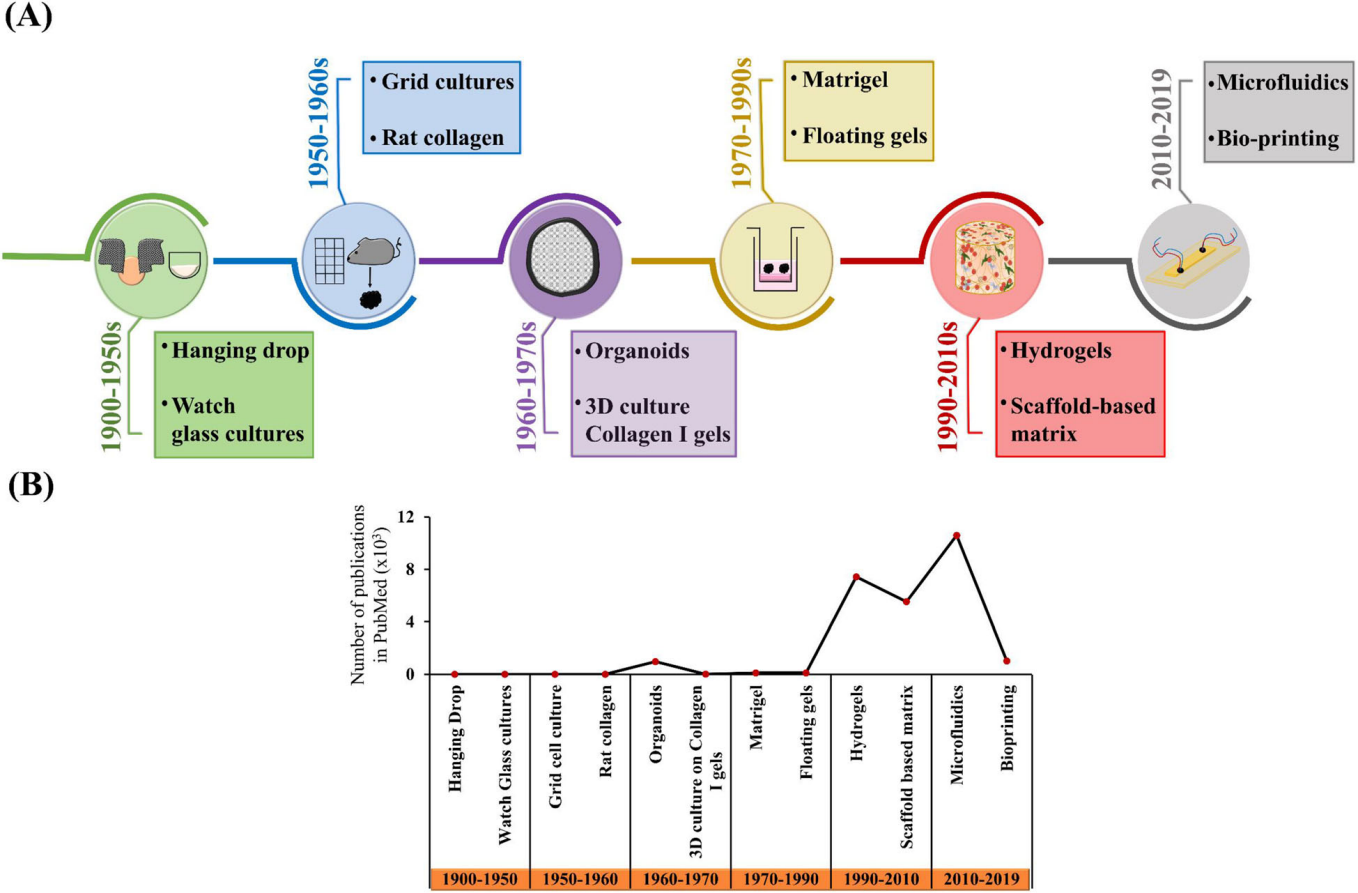
Timeline representing the evolutionary trend in the design of models that have established a third dimension to cell culture studies. **a** The various types of 3D models developed in each of the specified periods. **b** The number of publications reflecting the various 3D models as listed in PubMed over the previous twelve decades. (Search performed in November 2019)

Approaches to recreate the TME in 3D models have evolved considerably over time. From early prototype models to the current state-of-the-art systems, 3D model designs have advanced immensely with our increased understanding of TME physiology. With time, the models have increased in complexity and served to recreate even more in vivo like parameters. As illustrated in Fig. 3a, although the idea of utilizing the third dimension in cell culture research has been well-practiced since the early 1900s, interest has spiked significantly over the last few years [97], which is reflected by the increase in the number of publications employing 3D models (Fig. 3b). The hanging drop and watch glass techniques are some of the first 3D design models that provided an efficient way to use non-adhesive cell culture technologies on pre-coated plates. The hanging drop method is especially helpful in promoting the spheroid formation of cells without attaching to the plastic surface of the plate [98]. Several other prospective 3D platform designs have surfaced after the successful isolation of collagen from rat tail. Now, it is possible to fabricate designs mimicking macromolecular networks in which various types of cells, such as mesenchymal cells can be incorporated. With the capacity to study cell-extracellular matrix interactions, the diagnosis and treatment of various pathophysiologies such as fibrosis and cancer have greatly improved. 3D models prepared using high-quality purified collagen type I, provide researchers valuable in vivo like suggestions leading to ideas that were never considered before. The introduction of organoids and Matrigel® matrix also significantly helped evolve the 3D cell culture model blueprints. The steep increase in the number of publications that used these two platforms of study reveals the interest generated by these systems during the early days of 3D cell culture. The compact nature of innovative microfluidic platforms could be thought of as the organoid of this decade. Exceptional microfluidic device designs that challenge unanswered biochemical questions look promising to serve as prototypes for a new era of 3D in vitro models.

## Engineering TME hypoxia in 3D experimental models

While creating 3D experimental models to study cell-tissue interactions, the importance of using a prototype that aims to study TME hypoxia has always been a top priority. In order to fully understand the numerous consequences by which a low oxygen niche within the TME can affect tumor pathogenesis, several factors need to be concurrently embedded in a single 3D system. A hypoxic microenvironment could modify gene expression patterns in tumor cells [99], promote angiogenesis [100], increase chemoresistance [101], and support metastasis [102]. Altered cell metabolism under hypoxia, through stabilized HIF-1α, decreases cell proliferation [103]. As cell proliferation is nourished by an oxygen-enriched environment, a hypoxic TME results in a problematic fraction of cells that can survive the hypoxic stress and start exhibiting invasive characteristics [104]. While most of the cancer drugs are designed to target rapidly proliferating cells, tumor cells in a hypoxic niche evade the therapeutic effect of these drugs by demonstrating low proliferation [105]. Low oxygen can result in an acidic environment, relative to the pH heterogeneity inside the TME [106], which inhibits drug uptake rates in cells due to the impediment of molecule diffusion as a result of unnecessary cell membrane charging [107]. Most cancer drugs are weakly basic drugs that become ionized in the acidic hypoxia-driven TME and hence fail to exert their therapeutic effects [108]. Moreover, an altered ECM produces an additional hurdle for cancer drugs and cytotoxic immune cell infiltration into the hypoxic TME [26].

The effect of TME hypoxia on tumor cells has been studied extensively in 2D monolayer cultures [109]. These 2D hypoxia experiments are mostly performed by incubating cells in incubators or gas-chambers, where the amount of oxygen can be controlled as needed. Although simple, these models fail to achieve all of the experimentally relevant aspects associated with in vivo conditions. For example, a 2D model fails to recreate TME oxygen gradients and creates a polarized two-dimensional system of either high or low oxygen [110]. On the other hand, studying hypoxia in vivo has been difficult due to the high degree of oxygen tension variations among tissues [111] and the limited ability to identify chronic versus acute hypoxic factors within the TME [112]. As a result of these failures, researchers have sought multiple tools for designing 3D platforms to study tumor hypoxia. Some of the advances made in the hypoxic 3D cell culture model realm are outlined below.

### Spheroid and Organoid models

A well-explored route taken by researchers to mimic the hypoxic TME is the development of cell spheroids [93]. These models consist of a cohesively clustered module of tumor cells that somewhat recreates a tumor-like niche. The efficiency of cell spheroid as a model to study tumor hypoxia depends on several factors, such as spheroid size and culture media composition, which can be modified to predict viability and kinetic potential accurately [113]. Researchers can alter numerous factors related to TME surroundings like growth conditions to achieve desired results. Only those spheroid models that are in the diameter range of 200–500 μm are selected to be utilized for these studies [114].

The spheroid size can heavily regulate the efficient development of hypoxia within its core. A recent study [115] recreated the glioblastoma TME using a spheroid model containing three glioblastoma cell-lines (U87, U251, and SNB19). In the study, the researchers used the spheroid model to evaluate how glioblastoma cell gene expression responds to 1% oxygen tension and whether or not the cells could revert to their physiological genotype after the stress was removed. The goal of this work was to address the mechanism of how glioblastoma cells adapt to hypoxic and normoxic microenvironments and how the effects extend to critical health outcomes, including cell proliferation, metabolism, migration, metastasis, and angiogenesis. The spheroids were able to solve the primary challenges addressed in this study, including how TME hypoxia can reduce glioblastoma proliferation, migratory tendency, and how reversible these effects were once hypoxia was eliminated. Although the study did not adequately infer as to how long cultured cells might need to be exposed in hypoxic TME to make their hypoxia-driven pathological signatures irreversible, this study echoes the importance of designing a model that supports the genetic signature aspects responsible for switching between oxygen-stress related cell phenotypes.

Another study harnessed the power of spheroids to evaluate the role miRNAs play in driving tumor-initiating cell (TIC) or cancer stem cell activity within the oxygen-deprived tumor [116]. By utilizing spheroids, the authors consistently pooled an enhanced clonogenic capacity of different TIC cultures, which reflected how hypoxic conditions can actively assist the growth of various tumorigenic factors. Also, the use of spheroid cultures enabled the authors to achieve two critical experimental facets –the need for very few cells to achieve the relevant results and to perform the experiments in a setup that closely mimicked the in vivo outcomes.

Organoids are another advancement in 3D models that have been able to recreate a hypoxic TME. The use of organoids to study tumor hypoxia hallmarks have demonstrated the recreation of miniature arrays of hypoxic cell-derived, self-organizing, tissue-specific outcomes that mimic their in vivo counterparts. One such study highlights the use of an organoid model to recapitulate tumor hypoxia and stem cell heterogeneity [117]. In this study, the authors elucidate how patient-derived organoids can bridge the gap between classic in vitro and in vivo cultures. The attainment of in vivo like spatial orientation relative to the stem cell niche within these organoids is something classic models fail to exhibit and hence support a much more realistic and rational hypoxia niche. Also, these models allow multiple stem cell populations to grow, in an in vivo like heterogeneous manner. The authors demonstrated that the organoid-derived xenografts employed in this study were able to recapitulate single-cell phenotypes as present in the original patient tumors.

Cerebral organoids were studied to understand how injury related to oxygen deprivation can affect the health of human neuroprogenitor cell subtypes [34]. As it is critical to approach this problem by using a model that can support this complex cytoarchitecture, where the growth of different neuroprogenitor subtypes can simultaneously exist within a defined spatial arrangement, organoids were the platform of choice. To achieve hypoxic injury, the authors cultured the processed organoids in a hypoxic chamber under 3% oxygen culture conditions for 24 h before switching back to 21% oxygen culture conditions. Due to the cellular heterogeneity provided by these cerebral organoids, the authors were able to recreate a good approximation of how continuous hypoxia can affect the early stages of cerebral cortex development as compared to temporary oxygen deprivation under in vivo conditions.

One of the significant hurdles that researchers face while using organoids is to correctly engineer the cellular complexity in a controlled manner that linearly demonstrates a schematic assembly of organ function. Also, when studying tumor hypoxia using organoids, a major hindrance for researchers has been the limited capacity of oxygen deprivation because of inadequate surface diffusion. This results in incubated cell death over more extended culture periods. Despite the shortcomings, scientists have rigorously used the organoid platform to examine how the diseased organ can be targeted for better treatment regimens.

### Scaffold-based models

Biotech/biomedical companies, as well as academic scientists, have joined in the march to develop a perfectly suitable 3D scaffold that, while mimicking TME oxygenation profiles, can aid researchers’ design of improved chemotherapies. One such group at Brit Life Sciences developed the Cell-Mate 3D®, composed of natural hyaluronic acid and chitosan, which is individually responsible for providing a platform that exhibits features of the hypoxic TME [118]. Although the technology behind the development of Cell-Mate 3D is proprietary, it sheds light on the importance and the need for similar 3D models that can accommodate the TME oxygen content in a gradient-dependent manner for better therapeutic approaches. As an example, a bone marrow-derived 3D scaffold showed recreation of oxygen and drug gradients by manipulation of the scaffold height, with steeper gradients compared to other 3D platforms or 2D models [119]. As a result, this model allowed for the study of oxygen gradients on drug uptake and resistance. In another study, the importance of oxygen gradients to developmental and regenerative cellular processes like vascular morphogenesis and cancer cell health have been explored by designing and maneuvering a previously reported gelatin-based oxygen-controllable platform to recreate oxygen gradients necessary for predicting cellular functional responses within a 3D in vitro or in vivo niche [120]. The model reported in this study overcomes the hurdle of oxygen gradient generation while encapsulating tissue grafts in vivo via rapid oxygen consumption during the fabrication stage by a laccase-mediated cross-linking reaction. The model has tremendous potential to be used for various experiments that require real-time oxygen conditioning like the in vitro screening of small-molecule therapies. It can also be injected in vivo either in an acellular or cellularized state to test therapeutic outcomes. When performing in vivo experiments, the authors indicated that the hydrogel’s volume or the host tissue oxygen concentration could be pivotal for engineering the correct oxygen gradients. On a similar note, the contribution of hypoxic gradients has also been correlated to cancer metastasis in a study performed by Godet et al. [121]. The spheroid and organoid platforms used in this study demonstrated oxygen gradients. When incubated under 20% oxygen culture conditions, the oxygen content at the spheroid core was measured to be about 1%, while at the surrounding matrix niche was 11%. After 11 days in culture, the organoids demonstrated a drop in oxygen content from 14 to 1.7%. Both these models were composed of breast cancer cells that were permanently marked to reflect hypoxia. The authors leveraged these differences in oxygen to fate-map the resident hypoxic tumor cells. The results shed light on how hypoxia can influence several factors that perhaps play a role in promoting metastatic outcomes.

### Microfluidic models

Microfluidic 3D model platforms contribute to solid tumor hypoxia studies by supplementing a much-needed fluid-flow aspect achieved through cleverly designed micro-channels. Song et al. developed a microfluidic model that can systematically evaluate individual TME molecular factors and their relative downstream pathological effects, which is otherwise challenging to study in complex in vivo tumor samples [122]. In this study, the researchers measured the extravasation potential of different breast cancer cell lines after subjecting them to variable oxygen conditions, which include normoxic culture conditions reflected by incubation in 21% oxygen and hypoxic culture conditions reflected by incubation in either 3% or 1% oxygen. The in vitro assembly used in the study was composed of microvascular networks in a microfluidic chip. This model successfully reflected the importance of hypoxia dependent HIF-1α signaling in TME, and the microfluidic model was used to understand how HIF-1α knocked down variants of the breast cancer cell lines (MCF10A, MCF-7, and MDA-MB-231) can drive metastasis during cancer development and progression. Additionally, the model was very efficient in recreating known tumor hallmarks, including those affecting cell morphology and viability. The 3D model in this study overcame multiple problems reported in similar studies performed previously in 2D systems, e.g., where lack of proper visualization hindered evaluation of extravasation [123] and where the 2D nature of the study model failed to conclude the influence of CXCR4 expression in promoting cancer cell adhesion and transmigration [124]. The 3D model in this study reflected many well-known TME features like the loss of E-cadherin and the increase in vimentin expression. However, the model could not satisfactorily establish the mechanisms by which these factors were altered under oxygen-deprived conditions. Additionally, the model failed to conclusively determine a connection between the increased HIF-1α protein expression levels and TME invasiveness.

## 3D experimental models that address recreating TME-immune interactions

**Fig. 4:**
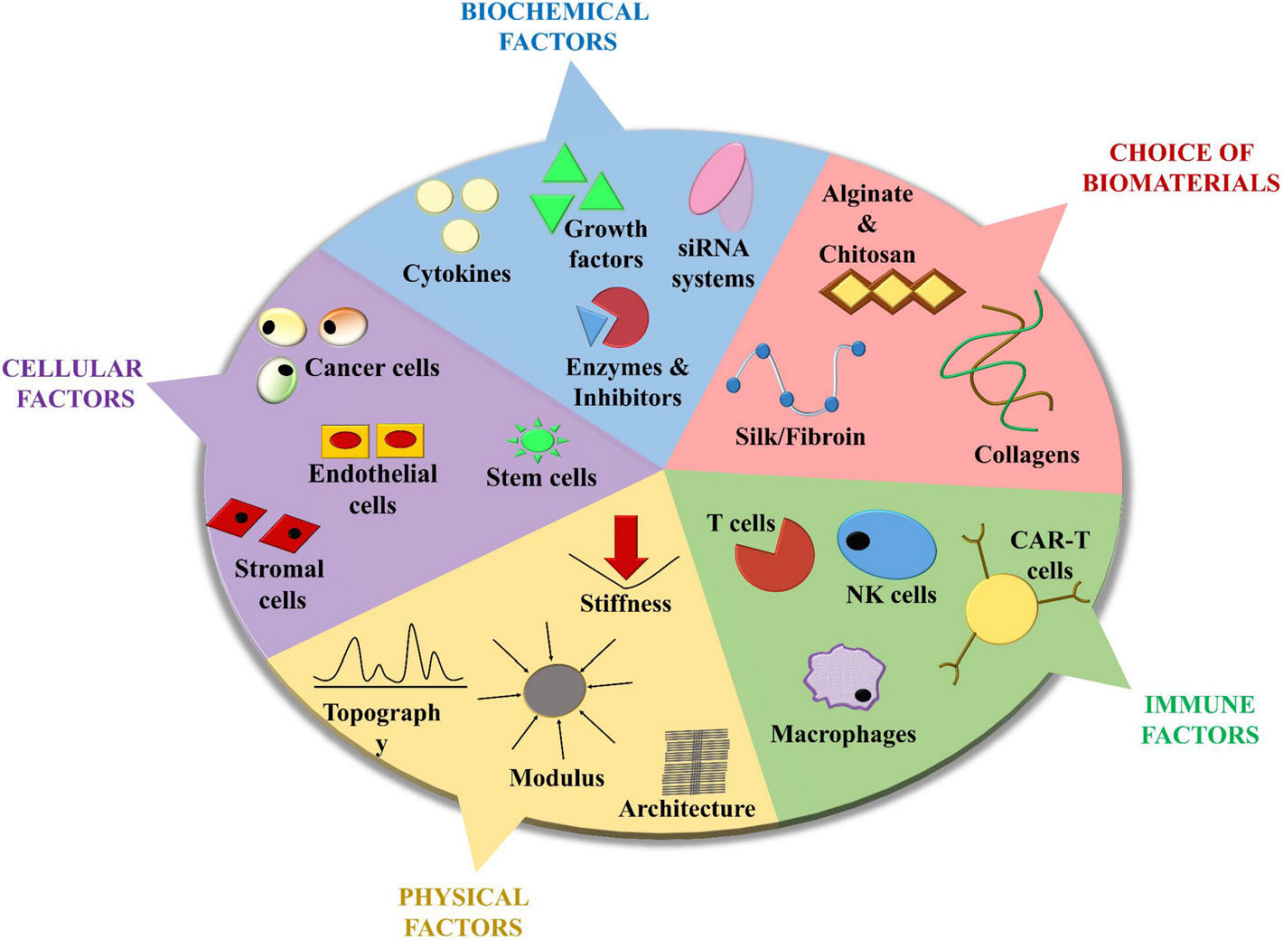
A pie chart representing the five more relevant hallmarks that define the design of an ideal 3D tumor platform that constitutes intrinsic tumor-immune interactions including: choice of biomaterials (e.g. alginate, chitosan, collagen and silk/fibroin); cellular factors (cancer cells, endothelial, stromal or stem cells); immune factors (T cells, NK cells, macrophages and MDSCs); biochemical factors (cytokines, growth factors, enzymes and inhibitors and microRNA systems); and physical factors (stiffness, topography, modulus and architecture). While each component is critical to efficacy, the goal is always to achieve a suitable balance between these five components while designing each 3D cell culture model

The need to develop dynamic TME models that allow researchers to study the extent of immune cell infiltration into the tumor corresponds to the demand to improve preclinical screening of immunotherapies. As the latest advancements in immunotherapy research are focused on the use of immune checkpoint inhibitors, these models would be essential to monitor the mechanisms by which TME influences immune evasion. Lately, the 2D models used to study tumor-immune interactions are being upgraded to 3D models as they help to reveal new immunotherapeutic perspectives [125]. Classic 2D models that have been used to study tumor-immune biology mostly employed permeabilized supports or Boyden chambers to investigate chemotaxis of immune cell migration and invasion [126]. Although these models have established interplay between numerous tumor-immune interactive aspects, they do not conclusively mimic the events that may challenge immune cell infiltrations into the tumor. Hence, there is a need to develop in vivo-like 3D multicellular models that seamlessly reiterate multiple tumor-immune aspects. The basis for an efficient 3D model that achieves this would depend on five critical hallmarks, as illustrated in Fig. 4: the choice of the biomaterial to create the 3D platform, the interplay of the various cellular heterogeneities, the addition of biochemical cues that would help maintain the homogeneous niche. These biophysical cues help retain the in vivo characteristics and the choice of immune cell types being selected for infiltration testing. The ideal 3D platform would need to be prepared by effectively balancing each of these hallmarks. Despite researchers modulating one or more of these five parameters: biomaterials: [26], cellular factors, biochemical factors, physical cues: [89] and immune system components: [127], very few have incorporated all five hallmarks into a single model.

A large number of tumor ECM components have been modified to construct the TME in 3D models, such as the use of biosynthetic collagen platforms [128] and fibrinogen based systems [119]. While these approaches have effortlessly recreated TME characteristics, scientists have also utilized many ECM-like materials as building block substitutes for creating 3D TME models. The choice of such an alternative material creates value in terms of availability and economic aspects. One such commonly used substance is silk fibroin protein procured from the cocoon of the silkworm *Bombyx mori*. Silk fibroin is being investigated extensively to produce an ideal TME building block for a 3D model [129]. The silk fibroin generates a non-immunogenic matrix after implantation, is highly maneuverable in terms of biodegradation, is stable in terms of robustness, and is abundantly available. Ultimately, the choice and combination of multiple parameters while constructing the platform, and the design blueprint, will determine the applicability and functionality of the 3D model. Some of the studies that address designing 3D approaches to recreate evasive tumor immune cell infiltration are listed as follows.

### Spheroid models

Many groups have been researching techniques to develop 3D immune-oncology models that reflect TME interactions with peripheral blood mononuclear cells (PBMCs). For example, a study performed by Sherman et al. fused 96-well permeable support in a low-attachment microplate system [130]. The hypothesis behind this design was that these permeable support systems would enable the evaluation of immune cell migration. Chemotactic response of human stromal-cell derived factor-1 (SDF-1α) on natural killer (NK)-92MI cells was tested by this study. Furthermore, they investigated how these NK-92MI cells would infiltrate into A549 (an adenocarcinoma lung carcinoma) cell-loaded spheroids that were developed in these low-attachment microplates. The most important takeaway from this study was that the net result of pairing a permeable support system with low-attachment microplates mimicked the entire PBMC homing mechanism, thus revealing how they can infiltrate tumor spheroids. The model’s conduciveness for NK cell infiltration into the tumor was evaluated using histology and confocal microscopy. The results indicated that the NK cells were not only able to infiltrate the simulated TME but were also successful in moving into the depth of the spheroid to interact with tumor cells. Models like this are critical in providing proof-of-concept evidence that highlights the importance of 3D models in recreating significant tumor-immune interactions. Eventually, the inclusion of non-cancerous cells in these models could further help in the understanding of how non-malignant tumor components may affect overall infiltration. Also, as in any spheroid system, the exact formation, size, and duration of multicellular spheroid cultures is cell line dependent, and a heavy emphasis needs to be given to optimizing cell-seeding conditions rather than letting their natural confluence exert therapeutic effects.

There is another interesting study that uses a spheroid platform (3D-3 culture), for assessing the variability in macrophage plasticity and evaluating how these differences can translate to the surrounding tumor microenvironment [131]. The 3D-3 culture model was created using an alginate microencapsulation method. The platform supported the growth of a cell cohort that included non-small cell lung carcinoma cells, cancer-associated fibroblasts, and monocytes in a well-defined co-culture setup. The authors demonstrated several tumor–immune facets frequently seen inside tumors, including the accumulation of extracellular matrix elements, cytokines, and chemokines over various study periods. The authors were also able to harness the infiltration of peripheral blood-derived monocytes into these 3D-3 spheroids that led to their eventual transpolarization into an M2-like macrophage phenotype, as confirmed by the expression of immune markers characteristic to tumor-associated macrophages.

### Organoid models

Modeling the tumor-immune microenvironment within organoids is of therapeutic importance because modeling a platform based only on peripheral immune population response alone does not lead to a clinically recognized translatable outcome for understanding checkpoint therapy mechanisms. Organoids are not adequate for reflection of the immune system in its entirety unless co-cultured with lymphocytes or other immune populations. For example, a study evaluated the usefulness of developing patient-specific autologous T cell – tumor organoid co-cultures that may help study tumor-immune interactions [132]. The authors set out two main objectives in this study: first, to understand whether tumor-reactive T cells can ideally be generated in a co-culture model comprising of peripheral blood lymphocytes (PBL) and matched tumor organoids; and second, if these tumor-reactive T cells would be functionally capable of killing tumor cells. The authors were able to conclusively achieve an almost 60% success rate in yielding tumor organoids that were highly expandable and cryopreservable. These patient-derived organoids successfully preserved the original tumor features from where they were sourced. Interestingly, the authors also showed that organoids that were acquired from major histocompatibility complex-I (MHC-I) deficient tumors were able to retain this attribute. The subjection of test tumor organoids to autologous tumor-reactive T cell populations resulted in reduced organoid size, significant apoptosis, and eventually reduced survivability of the organoid. Two potential applications from this study, as aptly highlighted by the authors, are: first, this platform can be utilized to study a diverse range of biochemical pathways that mediate tumor cell sensitivity and are critical targets for overcoming immunotherapy resistance. Secondly, the platform sheds light on the likelihood of creating organoids that can support the sustained generation of patient-specific T cell products. As therapeutic efficiency and patient-response to therapy can vary tremendously depending on gene diversity and multifactorial T cell responses, the patient-derived model reported in this study could be of substantial therapeutic importance.

In order to unravel the entire potential of organoid technology to support tumor-immune studies, it is essential to explore the possibility of engineering the tumor immune microenvironment within an organoid. A holistic model recreating immune infiltration within a patient-derived organoid system has been characterized [133]. This model incorporated a unique design of tightly integrated epithelial and stromal platforms, forming a compartment that is comprised of stromal components. The model was designed based on the principle that multi-hit tumorigenesis is achievable within the simulated TME through its air-liquid interface. More importantly, because of the stromal-epithelial aspect in the organoid, this model allowed in vitro modeling of tumor-immune cell interactions as opposed to those driven solely by PBMC populations. Although the model reported in this study reflected several in vivo like properties, questions of its translatability remain because the researchers employed surgically dissected samples to isolate the test populations without assessing significant parallel patient treatment controls, thus leaving gaps in the prospective correlations between simulated TME and patient immunotherapy responses.

A third approach that researchers have commonly taken to incorporate the immune-niche into organoids is by resecting tumor tissue that already contains the patient’s stromal and immune components. For example, in the study performed by Finnberg et al., the authors propose a resected platform that can support primary tumor cells and other associated tumor cells for up to forty-four days following isolation from the source [134]. The organoid cultures were also capable of sustaining the expression of tumor markers, as typically seen in patient tumors. The goal of this study was to use this patient-specific platform to immune profile blood and tumor tissues for the increased presence of various immunosuppressive entities in the presence of all tumor-associated components. The model also indicated that it might be functionally viable for over a month in culture. Some of the critical observations demonstrated by this platform include the comparative understanding of how cell populations from various sources may be maintained, i.e., despite a positive CD45^+^ expression, there was a reduction in observed CD3^+^ cells after 8 days of culture. Besides, growth variations in several populations of myeloid-derived suppressor cells, double-negative T cells, and monocytes were found to be sensitive to drug response after immune profiling patient tumor and blood using this model. Overall, the organoid system reported in this study promises to be an essential benchmark that could help reveal a broader perspective of how various immunosuppressive mechanisms regulate tumor growth and metastasis in a patient-centric manner. The model could also be used to create patient-specific therapeutic regimens that help overcome patient-to-patient drug response differences.

## The models that collectively address tumor hypoxia and immune evasion

Despite the importance of connecting tumor hypoxia and reduced immune surveillance within a single model, very few studies have achieved unifying these two hallmarks. On this note, there is a significant study that highlights the advantage of 3D cell cultures in reproducing hypoxia-dependent changes observed in the TME. In particular, hypoxia was shown to negatively regulate MHC expression in a HIF-dependent manner as demonstrated by lower MHC expression in hypoxic 3D models but not 2D tumor cell cultures in vitro. Downregulation of MHC class I expression is a hallmark of cancer and leads to cancer escaping recognition and rejection by anti-tumor T cells [135]. Furthermore, in a prior study from our lab, we recently showed that 3D models engineering physio- and pathophysiological oxygen levels allow for a better understanding of the role of oxygen availability in tumor-immune interactions. In particular, an oxygen-deprived environment was shown to recapitulate known breast cancer hypoxia characteristics such as reduced cell proliferation, increased extracellular matrix protein expression, and immune evasion mechanisms. Additionally, CD8^+^ T cell infiltration was significantly impaired under pathophysiological oxygen levels, and the inhibition of HIF or PD-L1 was able to re-sensitize breast cancer cells to cytotoxic T cells [136]. One more study that must be highlighted builds on the collective understanding that hypoxia can aggravate immune evasion in the physiological TME [127]. This research employed highly efficient chimeric antigen receptor T (CAR-T) cells, coupled with a microfluidic platform that accurately generated gradients of oxygen to mimic TME hypoxia. Immune cell infiltration into the 3D model was observed to vary significantly based on the oxygen content, reflecting the already well-understood series of evasive immune events that occur as a result of low oxygen within the tumor. As recreating an oxygen profile in a 3D model is essential to determining the exact tumor-immune evasive mechanisms, the model reported in this study could accelerate hypoxia-specific immunotherapies encompassing specific biochemical mechanisms for each immune cell type.

## Conclusions

The development of 3D ex vivo models is an area of rapid expansion, but the integration of immune cells in these cultures remains in its infancy. Further studies will warrant a better understanding of whether creating 3D in vitro models while mimicking tumor hypoxia and tumor-immune interactions would fare advantageous and therapeutically beneficial over classical in vivo animal models. Significant advances have been made in 3D tumor engineering toward development of the ideal TME model that may adequately accommodate multiple tumor properties. An ideal 3D TME model would function effortlessly by either being entirely inert from the patient immune variations or by implementing a personalized platform that is constructed using the patient’s cells. A 3D model that would bridge the gap between tumor hypoxia, immune evasion, and recreation of TME features as close as possible to in vivo conditions, must be suitable for all the different tumor cell types, without affecting inter-cell biology or the potentiation of immunogenic reactions. Although the advances have been noteworthy, all of the synthetic or biologically derived systems that have been fabricated with or without cancer cells have unfortunately hit a roadblock at some point in the developmental pipeline. The systems that have been developed in the presence of cellular growth are promising, but only from a proof of concept perspective. Also, the 3D models that have shown cellular growth have primarily been able to generate only specific tumor cell types: mutually exclusive populations of cancer, endothelial, stromal, or immune cells. Future studies that address the development of a system that can fuse all the different cell populations simultaneously are necessary. Results from several studies over the years have shed light on the understanding of solid tumor biology, thus forming the basis of TME modeling within a 3D in vitro niche. Despite extraordinary developments, most models recapitulate only some particular aspects of the TME; thus, further research and more holistic models are needed. As we advance in the field of 3D modeling of the TME, our methodologies and designs will hopefully become more superior. The synchronous blending of cellular growth, oxygen profile, immune cell interactions, and heterogeneity within the same 3D model would be a highly rewarding therapeutic advancement.

## Data Availability

Tumor H&E image datasets presented in Fig. 2 are available from the corresponding author on reasonable request.

## Abbreviations

TME: Tumor microenvironment
ECM: Extracellular matrix
H&E: Hematoxylin and eosin
HIF-1α: Hypoxia-inducible factor 1-alpha
PHD: Prolyl-4-hydroxylase
MDSC: Myeloid-derived suppressor cell
Treg: Regulatory T cell
Th17 cell: T helper 17 cell
TAM: Tumor-associated macrophage
NK cell: Natural killer cell
VEGF: Vascular endothelial growth factor
PBMC: Peripheral blood mononuclear cells
CD8: Cluster of differentiation 8
CAR-T cell: Chimeric antigen receptor T cell
PD-1: Programmed cell death protein 1
PD-L1: Programmed death ligand 1
TIC: Tumor-initiating cell
PBL: Peripheral blood lymphocytes
MHC: Major histocompatibility complex
